# Lower Cervical Chordomas: A Case Report and Differential Diagnosis

**DOI:** 10.1002/cnr2.70270

**Published:** 2025-07-09

**Authors:** Yosita Muenkaew, Artit Jinawath, Wichit Cheewaruangroj

**Affiliations:** ^1^ Department of Otolaryngology‐Head & Neck Surgery, Ramathibodi Hospital, Faculty of Medicine Mahidol University Bangkok Thailand; ^2^ Department of Pathology, Ramathibodi Hospital, Faculty of Medicine Mahidol University Bangkok Thailand

**Keywords:** cervical chordoma, immunohistochemical analysis, lower cervical neck mass, primary bone tumors, surgical treatment

## Abstract

**Background:**

Chordomas are rare tumors that arise from notochord remnants and are typically located in the axial skeleton. Chordomas arising in the lower cervical spine are rare.

**Method:**

We report a case of a 79‐year‐old man who presented with a lump on the left side of his neck, indicating an unusual presentation of a chordoma in the lower cervical region. This report describes the clinical, radiological, and histological results of this patient who sought medical attention for a left‐sided neck mass. Additional diagnostic tests, including immunohistochemistry and biopsy, confirmed the existence of a chordoma affecting the lower cervical region.

**Result:**

The precision of a diagnosis of cervical chordoma hinges on imaging study results and confirmation provided by histological examination, typically through biopsy and immunohistochemistry. The primary treatment for cervical chordoma is the surgical resection. Adjunctive therapies, such as radiation therapy and, in some cases, chemotherapy, may be used to manage residual disease or recurrent tumors.

**Conclusion:**

This case emphasizes the rarity of lower cervical chordomas, which present as a neck mass and are a diagnostic dilemma. Total excision is complex and relies on combining multiple disciplines including imaging, immunohistochemistry, and adjunct radiation therapy. Long‐term follow‐up and early detection are key to better outcomes and reduced recurrence.

**Level of Evidence:**

5.

## 1 Introduction

Chordomas originate from remnants of an embryonic structure known as the notochord, which assists in spinal bone formation during fetal development. During spine formation, notochord cells often disappear. However, small remnants of notochord cells continue to exist in some cases. Chordomas, which develop from these residual cells, are rare tumors primarily found in the axial skeleton. The sacral region (50%), spheno‐occipital area (35%), and spinal region (15%) are the most commonly reported anatomical locations for chordomas [1, 2]. They predominantly occur in patients aged 40–70, but they can also affect younger individuals, including children [3].

Radiological examinations are typically necessary for the problematic diagnosis of chordomas. After confirmation of the histological diagnosis, the primary treatment goal is generally considered to be complete surgical removal [2]. When feasible, adjuvant radiotherapy should also be administered. A lack of computed tomography myelography or magnetic resonance imaging (MRI) to help with the initial radiological evaluation often results in several misinterpretations, making chordomas extremely difficult to diagnose and frequently requiring multiple surgeries [4]. The limited information on lower cervical chordomas highlights the urgent need for more studies.

Despite advances in the understanding of chordomas, lower cervical chordomas with a presentation of a neck mass are uncommon, and diagnostic and treatment protocols are not yet clearly established. This case report contributes to the literature by reporting the clinical, radiological, and histological presentation in addition to the diagnostic evaluation and management strategies for this condition.

## 2 Case Report

The patient initially presented to Ramathibodi Hospital, Bangkok, Thailand, in January 2022. This case was retrospectively reviewed and reported in March 2024 following approval from the Human Research Ethics Committee, Faculty of Medicine Ramathibodi Hospital (MURA2024/369), by the ethical standards of the Declaration of Helsinki. Written informed consent was obtained from the patient to publish the case details.

**FIGURE 1:**
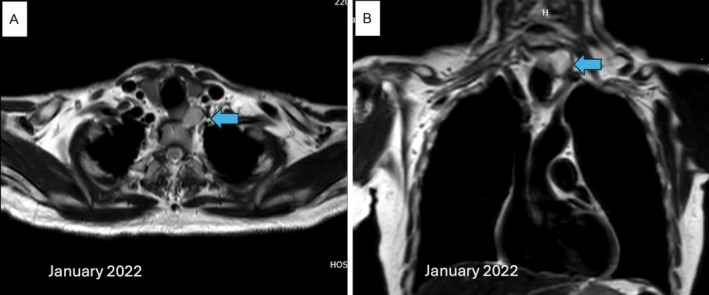
Preoperative MRI from January 2022 showing a thin‐walled cyst in the left paravertebral region at C7–T1 (arrows). (A) Axial and (B) Coronal planes.

A 79‐year‐old man was found to have a tumor on the left side of his neck with concerns about a gradual enlarging, which he had noticed developing over the past year. He reported no associated symptoms such as pain, dysphagia, or voice changes. He had undergone routine health check‐ups over the past 2 years (2020), during which no palpable neck mass or abnormalities were identified. The lesion was firm, fixed, and palpable on examination in the lower cervical region. The medical history and routine laboratory investigations were unremarkable, and no notable neurological deficits were identified on examination. MRI revealed a thin‐wall cyst of 30 × 22 × 24 mm on the left side of the paravertebral region at the levels of C7 and T1 (Figure 1). Moreover, the tumor lined the left lateral wall of the esophagus. Based on localization and the clinical picture, the tumor was mainly diagnosed as a slow‐growing lesion, that is, a cystic schwannoma, and not an esophageal duplication cyst.

A fine needle biopsy was performed under ultrasound guidance. The cytological analysis showed few neoplastic cells in loosely cohesive clusters and singles. The diagnosis showed evidence of the neoplastic process, the differential diagnoses of which were metastatic carcinoma and involvement of another type of neoplasm with foamy cells or apparent cell changes. Surgery was performed through an anterior transcervical approach in April 2022. Intraoperatively, a firm, well‐encapsulated mass was visible behind and beside the esophagus. The lesion was removed intact after being dissected from surrounding tissues and the level of C7 of the spine. The postoperative course was uneventful, with discharge on day four.

**FIGURE 2:**
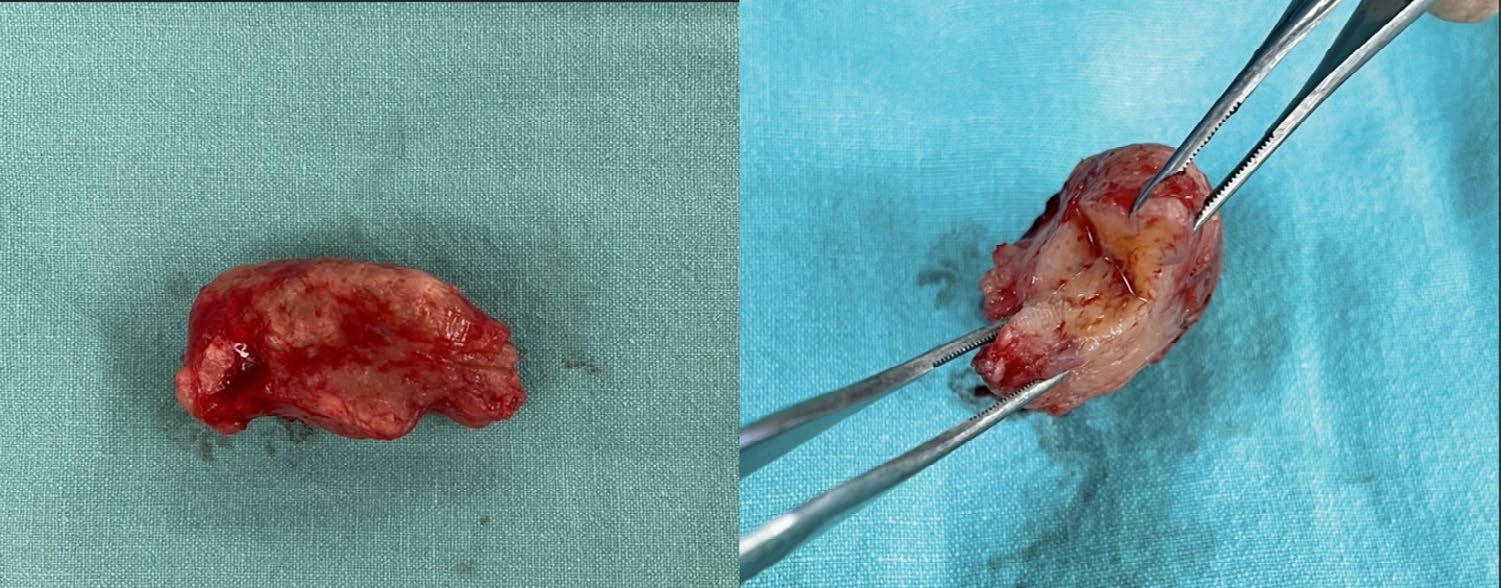
Gross appearance of the cervical chordoma.

**FIGURE 3:**
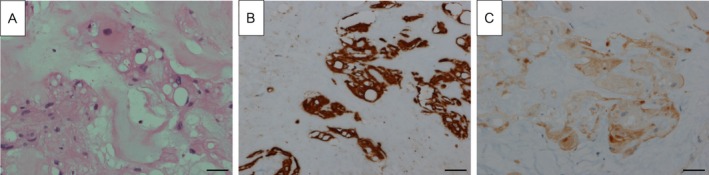
Microscopic image of characteristic chordoma histology: (A) Foamy cells (Hematoxylin and eosin, original magnification), (B) Diffuse cytokeratin AE1/3 staining, (C) S100 staining. Images acquired at 400× magnification. Scale bar = 10 μm.

Incidentally, the diagnosis of chordoma was made on histopathological grounds. Gross appearance featured several irregularly spaced, rubbery, tan‐brown pieces of tissue measuring 37 × 32 mm in total (Figure 2). Histology images indicated an epithelioid tumor, while differential diagnoses were chordoma and myoepithelioma (Figure 3A). Immunohistochemical analysis showed positive reactivity for AE1/AE3 (Figure 3B), S100 (Figure 3C), epithelial membrane antigen (EMA), CAM5.2, NSE, and Ki‐67 (< 1%) and negative reactivity for SOX10, P63, SMA, and calponin, which indicated tumors of a notochordal origin and confirmed the final diagnosis of chordoma.

**FIGURE 4:**
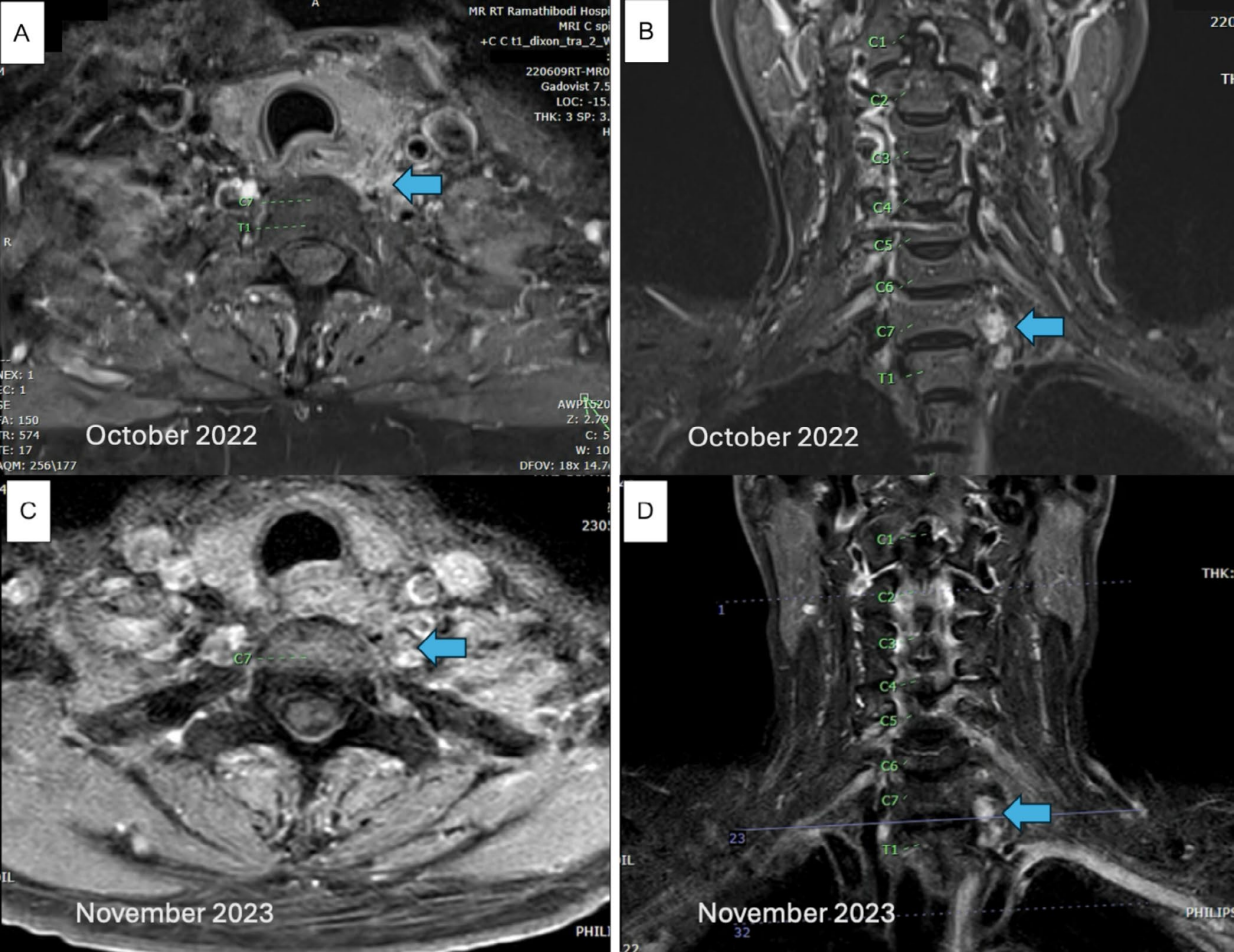
Six‐month follow‐up MRI showing a T1‐isointense, T2‐hyperintense lesion along the inferior border of the left C7 vertebral body, suggestive of residual tumor. (A) Axial T1‐weighted and (B) Coronal T2‐weighted images from October 2022. Follow‐up MRI from November 2023 shows no significant change in the 0.9 cm lesion, suggesting either residual tumor or post‐treatment changes: (C) Axial T1‐weighted and (D) Coronal T2‐weighted images.

Due to the presence of a tumor alongside the excisional margin, the patient was referred to an oncologist and radiotherapist for evaluation. The patient and family deliberated on the potential application of radiotherapy, both X‐ray and proton therapy. Even though the tumor was radioresistant, the purpose of radiation therapy was to reduce the risk of recurrence. Due to the expense, the patient chose the x‐ray radiotherapy. Therefore, on a five‐day‐per‐week regimen, the patient was treated with radiotherapy with a total dose of 6000 cGy administered in 30 fractions (200 cGy per fraction). Six‐and 18‐month postoperative follow‐up MRIs revealed residual tumors along the inferior border of the left‐sided C7 vertebral body (Figure 4). At the most recent follow‐up in March 2024, about 2 years after surgery, the patient remained neurologically intact with no evidence of disease progress. Clinical examination revealed no palpable cervical mass, neck pain, or neurological deficits, and motor strength was preserved (Grade V) in all muscle groups. No complications related to the disease or treatment were noted. The patient is scheduled for continued annual follow‐ups. A detailed timeline of the clinical course, treatments, and follow‐up is summarized in Table 1.

**TABLE 1:** Timeline of clinical events and management in a patient with lower cervical chordoma.

Date	Event description
2020	Routine health check‐up: No palpable neck mass detected
January 2022	Clinical presentation: Left‐sided neck mass and first MRI
March 2022	Fine needle aspiration performed under ultrasound guidance
April 2022	Surgical excision of the cervical mass
May–July 2022	Postoperative radiotherapy (6000 cGy in 30 fractions)
October 2022	6‐month postoperative MRI: Small residual lesion observed
November 2023	18‐month postoperative MRI: Stable residual lesion
March 2024	Latest follow‐up: No clinical or radiologic progression

## 3 Discussion

Chordomas are rare malignant tumors from notochordal remnants that often occur in the axial skeleton. The case in this study is unusual because it initially presented with a palpable neck mass, which is not typically found with chordomas. Though cervical spine involvement is familiar, the mass's fixed and complex nature, as well as the fact that it was close to the esophagus, made preoperative diagnosis particularly challenging [5]. This case supports the consideration of chordoma in the differential diagnosis of deep cervical masses, particularly given existing reports of such tumors presenting with variable neurologic involvement [4].

The extent of a chordoma impacts strongly on therapy and prognosis in the patient [6]. Chordomas are bone‐destructive malignancies that involve adjacent blood vessels and nerves, and presenting symptoms include pain and fatigue, especially in cases of spinal involvement. Early diagnosis, combined radiotherapy, and surgery are crucial to improve results [4, 7]. Due to the rare localization of the tumor and the fact that it is hard to diagnose, special attention should be given to this case. This article presents a complete review with a discussion of the case to aid clinicians.

### 3.1 Investigation

The current case highlights the extreme challenge of the preoperative diagnosis of cervical chordomas. Preoperative imaging showed a slowly growing lesion with an initial consideration of a cystic schwannoma or esophageal duplication cyst, which is in line with the nonspecific imaging presentation of chordomas that can mimic other tumors. Imaging of cervical chordomas, although suggestive, is rarely pathognomonic. Typical radiological appearances of spinal chordomas, such as destructive or lytic lesions, are commonly observed, although sclerotic changes can also be observed [8]. The imaging modality of choice is MRI due to its high ability to delineate the soft tissue extension of the tumor and its relationship to surrounding structures, including invasive or paravertebral involvement [9, 10]. Multiplanar MRI images are also useful for preoperative planning, providing valuable information about the extent and location of tumors. Chordomas typically appear as heterogeneous hypointense masses on T1‐weighted MRI, and gadolinium enhancement shows variable intensity patterns depending on cystic degeneration, osteophytes, and calcifications [11].

Fine‐needle aspiration biopsy yielded scanty neoplastic cells in this case, and a preoperative definitive diagnosis was challenging. This limitation is in concurrence with other reports in the literature, where the preoperative diagnosis of cervical chordomas is challenging due to their overlapping histological features with other neoplasms [12]. The histopathology of the current case, particularly the epithelioid tumor cells, initially raised a differential diagnosis of myoepithelioma. These findings underscore the diagnostic difficulty of chordomas, especially if they possess atypical histological characteristics, such as in the above‐reported cases. The differential diagnosis can include conditions such as chondrosarcomas, lymphomas, metastasis, multiple myeloma, osteomyelitis, primary nerve sheath tumors, and other paravertebral diseases, which can mimic the radiologic appearance of cervical chordomas.

### 3.2 Management

Surgery remains the treatment of choice for cervical chordomas [13], and a transcervical approach was utilized in this case to remove the tumor with negative margins. En‐bloc resection without spillage of the intraoperative capsule has been reported to be associated with reduced recurrence rates [14]. The pathology report indicated tumor cells at the surgical margin, and adjuvant radiotherapy was administered to reduce the risk of recurrence, maintaining the importance of negative margins in minimizing recurrence [4].

The histopathologic examination, with accompanying immunohistochemistry (IHC) results, helped establish the diagnosis of chordoma. AE1/AE3, S100, EMA, and CAM5.2 were consistent with chordomas, and the lack of staining for other markers such as SOX10, P63, and SMA excluded other neoplasms. These findings emphasize the importance of an imaging and IHC multimodal diagnostic approach for accurate diagnosis. Brachyury, not discussed in this article, is another helpful marker for chordomas.

Although chordomas are radioresistant, radiotherapy is appropriate if negative margins cannot be achieved [15]. This case is an indication of radiotherapy. Proton beam therapy, due to its precision and efficacy, would be most appropriate in irradiating tumors closely located in critical structures like the spinal cord and esophagus, as in this scenario [16]. While chemotherapy has no role in the first‐line treatment of chordomas, choices about treatment must be individualized by a group of specialists acting as a team, with the primary consideration being resection by surgery followed by irradiation.

### 3.3 Prognosis

Chordomas have a poor prognosis with local recurrence but do not metastasize, especially following incomplete resection [4, 6]. In this case, the tumor was not initially identified as a chordoma and thus was not entirely operated upon. Adjuvant radiotherapy controlled the residual disease despite the radioresistance of the cancer. The patient is symptom‐free with no progression 2 years later, which is a sign of successful treatment, although constant surveillance is necessary for potential late recurrence.

## 4 Limitations

The most significant limitations of this case are the diagnostic difficulty created by nonspecific imaging tests and histologic mimicry of other tumors, which create interpretive challenges. The complexity of the lower cervical anatomy made it difficult to achieve complete tumor resection, thereby increasing the risk for residual disease. The natural radioresistance of the tumor diminished the effectiveness of adjuvant treatment, making it essential to utilize a multimodal treatment and long‐term follow‐up to identify recurrence.

## 5 Conclusion

This case highlights the rare presentation of lower cervical chordomas and their rare presentation as a palpable neck mass, complicating diagnosis. A challenging preoperative workup is necessary due to the overlapping imaging and histological features. While radical surgery is ideal, its feasibility is tumor location‐dependent, thus rendering adjuvant radiotherapy necessary despite its limited effectiveness. Early diagnosis, multidisciplinary management, and long‐term follow‐up are essential to improve patient outcomes.

## Author Contributions

Y.M.: designed the case report and contributed to conceptualization, methodology, validation, investigation, formal analysis, writing – original draft, writing – review and editing, and project administration. A.J.: reviewed the pathology and contributed to investigation, resources, and writing – review and editing. W.C.: served as the primary supervisor of the case report, contributing to conceptualization, investigation, writing – review and editing, and supervision.

## Ethics Statement

The case report was approved by the Human Research Ethics Committee, Faculty of Medicine Ramathibodi Hospital, Mahidol University (MURA2024/369) and was conducted according to the highest ethical standard of the Declaration of Helsinki, ensuring the integrity of our research.

## Conflicts of Interest

All authors declare no conflicts of interest.

## Data Availability

The data that support the findings of this study are available on request from the corresponding author. The data are not publicly available due to privacy or ethical restrictions.
